# Supplementary material for: A protocol to simultaneously examine cardiorespiratory, cerebrovascular and neurophysiological responses inside a hypobaric chamber
Source: PLoS One. 2024 Oct 24;19(10):e0312622. doi: 10.1371/journal.pone.0312622 (PMC11500867; doi:10.1371/journal.pone.0312622)
Supplement: S1 Text — (PDF) [file pone.0312622.s001.pdf]

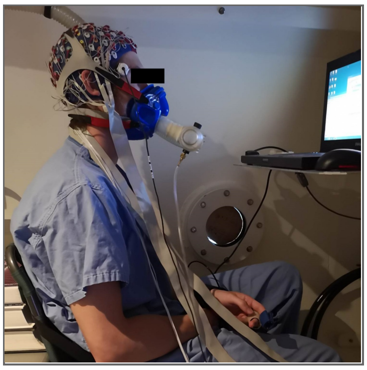

A Protocol to Simultaneously Examine Cardiorespiratory, Cerebrovascular and Neurophysiological Responses Inside a Hypobaric Chamber

Evan A. Hutcheon[1], Sherri Ferguson[2], Victoria E. Claydon[1], Urs Ribary[3], Sam M. Doesburg[1,4]

[1]Department of Biomedical Physiology and Kinesiology, Simon Fraser University, Burnaby, British Columbia, Canada;
[2]Environmental Physiology and Medicine Unit, Faculty of Science, Simon Fraser University, Burnaby, British Columbia, Canada;
[3]Department of Psychology, Simon Fraser University, Burnaby, British Columbia, Canada;
[4]Institute for Neuroscience and Neurotechnology, Simon Fraser University, Burnaby, British Columbia, Canada

Evan Hutcheon

Protocol Info: Evan A. Hutcheon, Sherri Ferguson, Victoria E. Claydon, Urs Ribary, Sam M. Doesburg . A Protocol to Simultaneously Examine Cardiorespiratory, Cerebrovascular and Neurophysiological Responses Inside a Hypobaric Chamber. **protocols.io** https://protocols.io/view/a-protocol-to-simultaneously-examine-cardiorespira-c46cyzaw

ABSTRACT

We describe a protocol to examine neurophysiological (electroencephalography, EEG), cerebrovascular (ultrasound assessments of middle cerebral artery blood velocity, MCAv) and cardiorespiratory (blood pressure, oxygen saturation, end-tidal gases, respiratory rate) responses inside a hypobaric chamber. This procedure aims to standardize the methodology in experiments conducted within a hypobaric chamber such as comparing normobaric and hypobaric hypoxia. This is important because current understanding of relationships between neurophysiological activity, and cerebrovascular and cardiorespiratory responses under varying environmental conditions remains limited. This procedure combines simultaneous neurophysiological, cardiorespiratory and cerebrovascular evaluations, allowing a comprehensive understanding of electro-neurophysiological activity. Our protocol requires an hour and a half of equipment setup, 1-1.5 hours of participant set-up, and 30 minutes of experimental data collection. Since multiple simultaneous physiological recordings, including EEG in this environment, can be fraught with pitfalls, we also provide practical considerations for experimental design and recording setup. Advanced knowledge of hypobaric chamber operation is required, alongside expertise in EEG and transcranial Doppler ultrasonography. Following our procedure one will acquire simultaneous recordings of neurophysiological, cerebrovascular and cardiorespiratory data.

GUIDELINES

Participant Considerations

The study protocol should be explained to the participants so they can make an

MANUSCRIPT CITATION:
Hutcheon, E. A., Vakorin, V. A., Nunes, A., Ribary, U., Ferguson, S., Claydon, V. E., & Doesburg, S. M. (2023). Associations between spontaneous electroencephalogram oscillations and oxygen saturation across normobaric and hypobaric hypoxia. Human Brain Mapping, hbm.26214. https://doi.org/10.1002/hbm.26214

Hutcheon, E. A., Vakorin, V. A., Nunes, A. S., Ribary, U., Ferguson, S., Claydon, V. E., & Doesburg, S. M. (2023). Comparing neuronal oscillations during visual spatial attention orienting between normobaric and hypobaric hypoxia. Scientific Reports, 13(1), 18021. https://doi.org/10.1038/s41598-023-45308-8

Created: Nov 16, 2023

Last Modified: Nov 16, 2023

PROTOCOL integer ID: 91044

informed decision regarding participation, and to ensure they understand the list of exclusion criteria and their importance. We suggest a relatively long list of exclusion criteria (S2). These exclusion criteria incorporate factors that might influence participant safety or comfort during altitude exposure such as pregnancy, smoking, respiratory disease, claustrophobia, nasal congestion, use of anti-hypertensive medication, anemia, psychiatric or neurological disorders, and any other medical conditions not compatible with altitude exposure. We also excluded participants with beards, in case this influenced the fit of the breathing mask, and individuals such as divers or pilots who may have developed physiological adaptations to hyperbaria or hypobaria that could influence their responses to the test. Excluding divers is also a safety concern, as it is not safe to dive prior to high-altitude exposure. Participants were included if they had normal or corrected vision.

There is a need to include more females in future studies, as there is a substantive lack of data on sex differences in responses to normabric hypoxia (NH) and hypobaric hypoxia (HH), with a recent review identifying that out of 13 studies, with a total of 153 participants, only 6 participants were women (Coppel et al., 2015). This is particularly important as there are reports of sex differences in EEG responses to NH (Rice et al., 2019). When including testing of female participants, care should be taken to ensure natural cycling women are tested at the same phase of the menstrual cycle as the associated hormone changes may influence test results (Das, 1998; Hayashi et al., 2012; Lee et al., 2003).

Technical Considerations

To ensure accuracy of measurement of end-tidal gases, the infrared gas analyzer (e.g., O2CAP) should be calibrated before use with gases of known concentration that span the range of end tidal gases expected for the protocol. For our protocol, we modified a nasal cannula by removing the bifurcation piece to enable attachment to the modified non-rebreather valve

for side stream sampling of end-tidal gases, with the other end connecting to the infrared gas analyzer. Care must be taken to ensure that the modified nasal cannula connecting the modified non-rebreather mask to the infrared gas analyzer is securely attached, as if a leak occurs the measured end-tidal gases will not be accurate.

When induced barometric pressure changes are expected, the modified nasal cannula should be securely attached to the modified non-rebreather mask first, with the other end only attached to the infrared gas analyzer once at the target pressure. We suggest this is done for all tubing, including for the blood pressure monitoring device and the tubing connecting the mask to the air reservoir bag. Once depressurizing is complete the tubing can be re-inserted. This is done as any gas in the equipment needs to be equalized with the ambient pressure during HH to avoid damage due to gas expansion on ascent.

It is important to explain to the participant that speaking can potentially disrupt the seal on the mask, and disrupt the end-tidal measurements. The participant should only speak if they are feeling unwell or voicing other concerns. Reassure the participant it is okay to speak if they are feeling unwell.

The Portapres blood pressure cuff and SpO_2 finger monitor are both sensitive to low finger temperatures. Depending on the temperature, you may have to cover the

participants arm, or even the participant, with a blanket to ensure accurate recordings. If the participant has painted nails the SpO_2 monitor may not be accurate, and the SpO_2 monitor might have to applied on another location (e.g., toes, ear) or have the participant remove the nail polish.

The inside hypobaric safety attendant will also manually adjust the airflow (Figure 2) in the back of the hypobaric chamber to match the participants breathing rate and be certain that the breathing gas reservoir bag (Figure) never collapses on itself or becomes too full. Participants breathing rate will vary between participants and may increase and/or decrease over the course of any hypoxic stimuli.

Ethical considerations

Participants should have ample time to review the informed consent information prior to making a decision about participating. The informed consent should include a list of potential risks associated with pressure changes in a hypobaric chamber and exposure to hypoxia. Ideally, participants should have an opportunity to familiarise themselves with the study expectations and visit the hypobaric chamber (Figure 1) prior to testing. During this familiarisation session they should be introduced to the study team, equipment and instrumentation, expectations, and any emergency procedures. This also provides a convenient opportunity to screen for eligibility and complete any relevant forms, including the consent form, and medical questionnaire (S2).

Design considerations

The primary design consideration is the simulated altitude at which the study is to be conducted. Once the altitude has been chosen, then a hypoxic gas mixture with an equivalent partial pressure of inspired oxygen to the altitude should be calculated. Here we show how we came to calculate our equivalent hypoxic doses. We selected a desired equivalent altitude of 3962 m for the HH condition to ensure a robust, but safe, desaturation that has relevance to the aviation industry. We utilized an altitude-pressure conversion table based on the United States standard atmosphere (Brombacher, 1936) to determine the barometric pressure of our target altitude of 3962 m (equivalent to a pressure of 464 mmHg).

The alveolar air equation (Richard & Koehle, 2012) was then utilized to find a hypoxic gas mixture (NH) that was equivalent to the reduction in atmospheric pressure used in our HH condition. First, we determined the partial pressure of inspired oxygen (PiO_2) during the HH condition, where P_B is the barometric pressure, P_{H20} is the partial pressure of water vapor in air, and FiO_2 is the fractional concentration of oxygen in air (0.209):

To calculate the percentage of oxygen required to simulate the desired PiO_2, we used a similar approach:

Based on these calculations, the PiO_2 for NH and HH were equivalent at 87.2 mmHg. For the NN condition the PiO_2 was 142.5 mmHg (based on a barometric pressure of 729 mmHg in our chamber, located 365 m above sea level).

These calculations could readily be adapted for different hypoxic doses as needed. We did not have participants breathe 100% oxygen during the depressurization to our target pressure. Accordingly, participants became slightly hypoxic during the depressurization, and started the experiment slightly hypoxic. If this is not desired, we suggest that participants breathe a 100% oxygen mixture during the depressurization to prevent them becoming hypoxic during the depressurization to the target altitude. If this approach is adopted, then for the NN and NH conditions participants should also be breathing 100% oxygen as part of the experimental blind. Another option is to not have participants breathing 100% oxygen during the depressurization in the HH condition, and have participants begin the experiment already hypoxic. In this case, for the NH condition participants should breathe the hypoxic gas mixture for the time it took to depressurize in the HH condition, or until their SpO_2 has stabilized to ensure an equivalent stimulus.

Immediately prior to the experiment participants should be exposed to a slight depressurization to test if participants can equalize their ears (Figure 3). Depressurize the chamber to 630 mmHg (1554 m) and then repressurize it back to ambient pressure to make sure that the participants can equalize their ears to prevent tympanic membrane rupture. We recommend this is done in all 3 conditions as part of the experimental blind. Remind the participant to signal if they feel they cannot equalize and want the depressurization to stop. While the chamber is depressurizing and pressurizing it is imperative that the inside hypobaric chamber safety attendant pays attention to the participants so that they can inform the outside chamber operator to stop depressurization or pressurization if the participant signals that they cannot equalize their ears.

If masks are used to deliver targeted inspired gases, participants should wear the mask in all three conditions as part of the experimental blind. We required a mask in the NH condition for delivery of the hypoxic gas mixture, and therefore required the participants wear a mask for the other conditions. Participants may hyperventilate if they are not used to wearing a mask. We recommend that studies have participants wearing the mask for 20 minutes before beginning the experimental paradigm so that they become used to breathing with the mask. Conversely, the hypobaric chamber could be flooded with nitrogen in the NH condition to create the hypoxic gas mixture within the chamber so that masks are not required (participants would

then breathe the ambient air within the chamber for all three conditions).
As part of the experimental blind, employ a depressurization and pressurization at the end of the NN and NH conditions to create a sensation of pressure change. This should occur right at the end of the task during NN and NH and take the same amount of time as pressurization during the HH condition. For our hypobaric chamber and pressure of choice, the pressurization back to ambient values was roughly three and a half minutes. During this time, we depressurized the chamber to 570 mmHg (2360 m) and then immediately pressurized the chamber back to 727.5 mmHg (366 m; which was the pressure at which our testing facility was located).

MATERIALS

Equipment

The equipment needed for this protocol will be listed in general, alongside the exact equipment we utilized.
• Electroencephalography recording equipment (used here: 64-channel Active Two Biosemi system, Biosemi, Amsterdam)
• Pulse oximeter (used here: Nonin 7500 Pulse Oximeter, Nonin Medical Inc, Plymouth, Minn)
• Infrared gas analyzer for end-tidal gases (used here: O2CAP; Oxigraf Inc., Mountain view, CA, USA)
• Beat-to-beat finger blood pressure measurement device (used here: Portapres model 2, TNO-TPD Biomedical Instrumentation, Amsterdam, The Netherlands)
• Analog to digital converter for cardiovascular measures (used here: sampled at 1kHz Powerlab 16/30; AD instruments, Colorado Springs CO, USA)
• Transcranial Doppler (TCD) system (used here: 2-MHz ultrasound probe DWL Doppler-Box; Compumedics)
• Hypobaric chamber (used here: Our hypobaric chamber was built by Perry Baromedical. The chamber is a triple-lock, multiplace Class "A" Hypo/Hyperbaric complex that contains an entry lock, main lock, and wet pot. Note that the wet pot, located beneath the chamber, was not used in the study and accordingly the hatch depicted that leads to it was closed (Figure 1). The chamber has a maximum working pressure capacity of 30 ATA (445 PSI) and a minimum working pressure of 0.01 ATA (0.15 PSI). The overall length, width, and height of the chamber are 7.3 m, 3.86 m, 2.2 m, respectively.)
• Reusable mask with straps (used here: 7450 V2 series reusable mask with mask adapter and headgear, Hans Rudolph Inc.)
• 2-way non-rebreather modified to allow for end-tidal measuring (used here: 2700 two -way valve with body style saliva, Hans Rudolph Inc.) (Figure 2)
• Separate mask connected to 100% oxygen inside chamber in case of emergency
• Compressed gas (use here: 100% oxygen and a 12.8% oxygen hypoxic gas mixture)
• Ear protection suitable for use during pressure changes (vented).

Software

Three laptops are required. One laptop with physiological data acquisition capabilities on which we utilized LabChart (version 8) software to record the cardiovascular and respiratory data. A second laptop with appropriate software presented the task paradigm (we utilized presentation software version 20.112.04.17). A third laptop recorded EEG data (we utilized Biosemi data acquisition software; actiview702-lores).

SAFETY WARNINGS

It is imperative to consider safety features for this experimental paradigm. It is a requirement for hypobaric chambers to have a fire suppression system that must be operational. The participant should

know where it is and how to activate it should an emergency occur and the hypobaric chamber safety attendant is incapacitated. To mitigate any risk of fire (of concern if the chamber is also used as a hyperbaric chamber) it is best practise to change into scrubs and not wear shoes while inside the chamber as the fire risk is exacerbated by clothing with flammable fibers and oil on shoes. If experimental laptops have lithium-ion batteries they must be removed before bringing them into the chamber and powered from the chamber electrical source in consideration for fire safety.

Before the first testing day instruct the participant that they are to have a light meal, and to drink a large bolus (500 ml) of water before each recording session to prevent syncope (Claydon et al., 2006; Mathias & Young, 2004; Parsons et al., 2022; Schroeder et al., 2002).

It is important to explain the possible risks of the procedure, in particular the risk of middle ear barotrauma. Participants should be familiarised with the Valsalva and Toynbee maneuvers to ensure they can effectively equalize their ears. The Valsalva maneuver is performed by a forceful expiration against a closed airway which is usually done by the participant pinching their nose and closing their mouth while expelling air out as if attempting to blow their nose (Hidir et al., 2011). The Toynbee maneuver involves the participant pinching their nose shut and swallowing a few times (Hidir et al., 2011). Remind them that if they have trouble equalizing their ears, or in general feel unwell during the depressurization, they are to let the hypobaric chamber safety attendant know. Our lab utilizes simple hand signals to ensure effective communication irrespective of background noise or the use of facemasks that may impair speech. For example, a closed fist hand signal indicates that the participant cannot equalize their ears. Once this signal is given, the hypobaric chamber will immediately stop depressurization/pressurization and allow the participant another attempt to equalize. If the participant is unsuccessful in equalizing the chamber pressure will be adjusted to allow the participant to equalize the pressure in their ears before continuing with the pressurization/depressurization.

Have the participant keep both feet planted on the ground. This is done to not restrict blood flow to the lower limbs, which may affect tissue off gassing and lead to a “pins and needles” sensation from restricted blood flow that can be mistaken as a sign of decompression sickness.

Remind the participant to let the inside hypobaric chamber safety attendant know if they are feeling unwell during the experiment and wish to terminate the experiment and be put on 100% oxygen. Let them know that there is a risk of syncope (both hypoxic syncope or vasovagal syncope due to them sitting still and/or being nervous with the experiment) (Hanna, 2014; Westendorp et al., 1997) and go over the pre-syncope warning signs such as “seeing stars”, feeling lightheaded, vision starting to fade, suddenly feeling hot, and generally feeling unwell.

Let the participant know to communicate any of these symptoms to the hypobaric chamber safety attendant immediately, at which point they will be given 100% oxygen to breathe, and the experiment terminated. Our experimental task has a break section every three minutes. During these breaks participants should flex/move their legs to minimise blood pooling to their legs and predisposing to a syncopal episode (Hanna, 2014). Advise the participant that in addition to criteria for stopping the test if they feel unwell, there are also physiological cut-offs (e.g., for our experiments we use SpO_2 continuously below 80% and systolic blood pressure drop of 40 mmHg as cut-offs), that if exceeded will result in them being placed on 100% inspired oxygen as a safety precaution. It is important that the set up allows for the inside hypobaric chamber safety attendant to monitor the SpO_2 and blood pressure to ensure compliance with any physiological cut points. Reassure the participant that they will still receive any study-related honoraria if the test is terminated because they meet safety endpoint criteria. This is important to prevent participants from attempting to conceal their symptoms for fear of jeopardising any honoraria or payments.

ETHICS STATEMENT

The study was approved by the Simon Fraser University Research Ethics Board and all participants provided written informed consent.

BEFORE START INSTRUCTIONS

Make a LabChart file with the relevant formulae for calculating the physiological variables. Ensure that for the HH condition any end-tidal carbon dioxide calculations are adjusted to account for the lower ambient pressure. Create a randomized participant testing schedule.

1 Bring all equipment into the hypobaric chamber (Figure 1) and set it up: experimental task laptop, EEG recording computer, physiological measures recording computer, capnograph, transcranial doppler (TCD) probe, masks, analog to digital converter, oxygen saturation (SpO_2) monitor, Portapres and have the EEG materials (gel, electrodes, cap) ready for the recording session. For our protocol, we had to cut a nasal cannula before the bifurcation so that we could attach it into the modified non-rebreather valve, with the other end connecting to the capnograph.

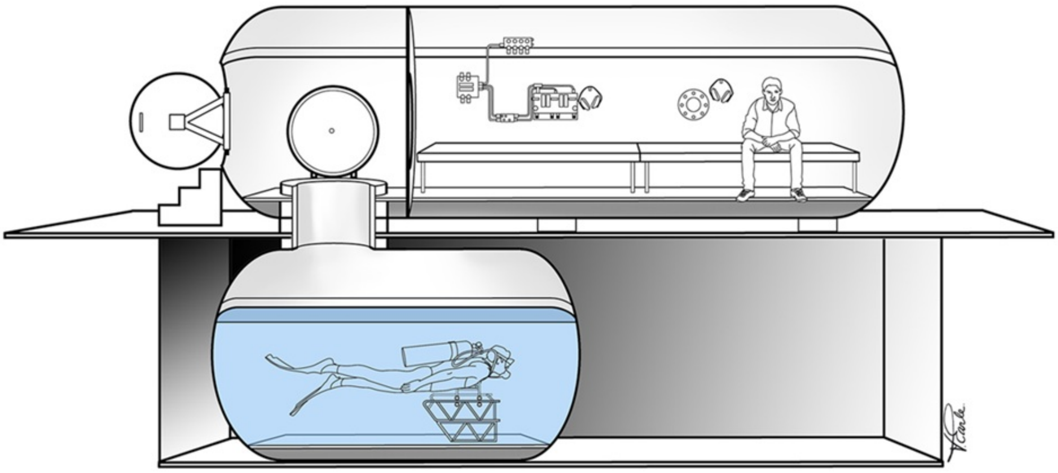

Figure 1. A schematic of the hypo/hyperbaric chamber in which we performed our three exposures. The bottom part of the chamber can be utilized for diving studies; however, for our study that section of the chamber was closed. All recordings took place in the main lock of the hypobaric chamber, where the figure is sitting.

2 Go over the experimental procedure once more with the participant. Provide them a private space to change into scrubs.

3 Measure the participant’s head circumference and put on an appropriately sized EEG cap. If desired the middle cererbral artery blood velocity (MCAv) Doppler signal can be identified at this stage, with the optimal settings and location indicated with skin marking pen, or alternately, later during step 11.

4 Apply the electrode gel and EEG electrodes to the participant, including the extraocular electrodes. The TCD probe will be attached on one side of the face, so insert the extraocular electrodes on the other side of the face. Place one vertical extraocular electrode above the center of their eye and the horizontal extraocular electrode 1.5 cm from the lateral canthus of the eye. Ensure adequate electrode gel applied for optimal signal acquisition. If necessary, abrade the skin at the electrode site to improve the signal quality.

5 Once the EEG has been applied, attach the cables for the electrodes to the EEG amplifier and check the electrode offset/impedance that all electrodes are below the cut-off values (this will vary depending on the type of EEG system).

6 Measure the participant’s middle phalanx of their left middle finger and attach the relevant Portapres finger cuff for their finger size ensuring optimal sensor positioning. Input the participant’s sex, age, weight, and height into the Portapres. Wait a few minutes recordings to stabilise, and confirm reading accuracy with a manual blood pressure reading with a sphygmomanometer. If hands are cold apply a warm blanket to optimise signal quality.

7 Remove any coloured nail polish if applicable. Attach the SpO_2 finger cuff on their left index finger. Again, if hands are cold apply a warm blanket to optimise signal quality.

8 Plug the loose end of the cannula into the capnograph gas analyzer. Remind the participant that speaking can disrupt the measurements, and to only speak if they are feeling unwell.

9 Help the participant with putting the mask on, and make sure that the straps of the mask do not shift any of the EEG electrodes. Once the mask is in place do a mask fit check by having them inspire and expire with maximal force while the valve of the mask is closed with the participants hand (Fikenzer et al., 2020). Carefully inspect the fitting for the absence of any indication of leaking such as noise (e.g., whistling, airflow) or visually seeing a break in the seal with lateral airflow. It is imperative that the participant's mask has a good seal. Adjust until optimal fit is achieved. Do not attach the regulator to the valve of the mask yet.

10 Add ultrasound gel to the TCD probe and carefully put it on the area marked before (step 4), being careful to not change its angle of insonation. Note that step 11 could be omitted in theory, with signal acquisition reserved until the present step – however we found having previously identified the optimal position and settings for location of the Doppler signal made it easier to find the signal once the participant was instrumented. This additional step improved the participant experience, as prolonged periods optimising signal acquisition inside the chamber with the EEG cap, mask and all the recording instruments can become uncomfortable for the participant. Once the MCA is located, optimize the signal quality with the depth, gain, power and sample window settings (Purkayastha & Sorond, 2013). Tuck the probe arms under the straps and EEG cap to secure it in place. A fabric headband is preferred as the probe may slip if inserted under the EEG cap, and therefore you may lose the angle of insonation and not maintain a reliable signal.

11 Once all the recording equipment is setup on the participant (Figure 2), the inside hypobaric chamber safety attendant will let the outside hypobaric chamber operators know to begin depressurization. Depressurize the chamber to 630 mmHg (1554 m) and then repressurize it back to ambient pressure to make sure that the participant can equalize their ears.

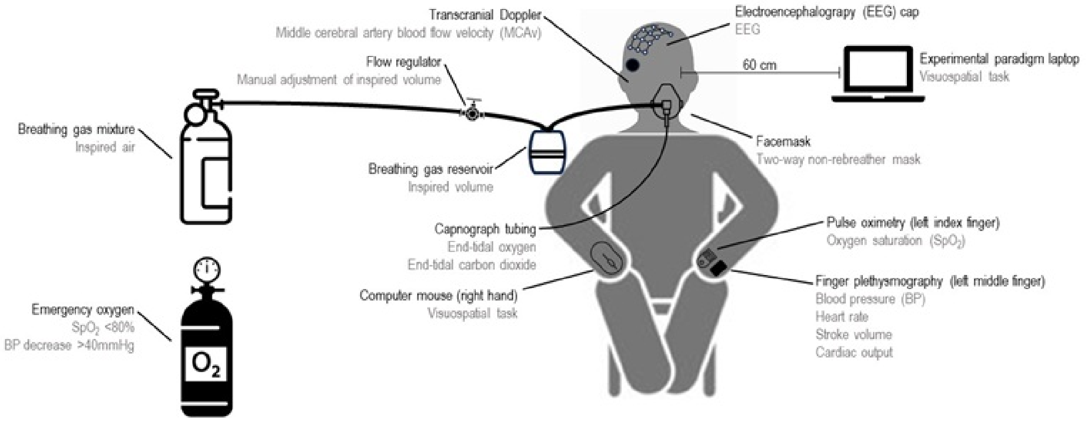

Figure 2. A picture showing schematic of our experimental set-up. The participants sat upright with their eyes 60 cm from the computer screen. They had a 64 channel EEG cap on, with a TCD ultrasound probe over the transtemporal window and held in place under the EEG cap. The participant wore a mask with a modified non-rebreather valve that allowed for side stream sampling of end-tidal gases. The participants left index finger had an SpO_2 monitor and their left middle finger had a finger cuff for collecting blood pressure data. The inside chamber safety attendant controlled the flow regulator making sure that the breathing gas reservoir bag never collapsed or became too full. Emergency oxygen was available if needed.

12 Continue with the experiment if the participant was able to equalize their ears. If the participant had trouble with equalizing their ears, either terminate testing at this time, or consider a second attempt, where you depressurize and pressurize the chamber at a slower speed. If this does not facilitate equalization of the participant's ears, terminate the test for safety. As part of the experimental blind, refer to each condition as the high altitude condition, and let the participant know this ahead of time. If you are in the HH condition the chamber will actually depressurize to the required pressure (for our experiment this was 464 mmHg (3962 m)), if you are in the NH and NN conditions then the vacuum pump will be on but all the valves will be open, so the participants experience the sounds of depressurization, but no change in pressure occurs (Figure 3). For reference, the depressurization to 464 mmHg (3962 m) took three and a half minutes on average (Figure 3) in our experiment. For the NH and NN conditions run the vacuum pump for three and a half minutes with the chamber door closed as part of the experimental blind.

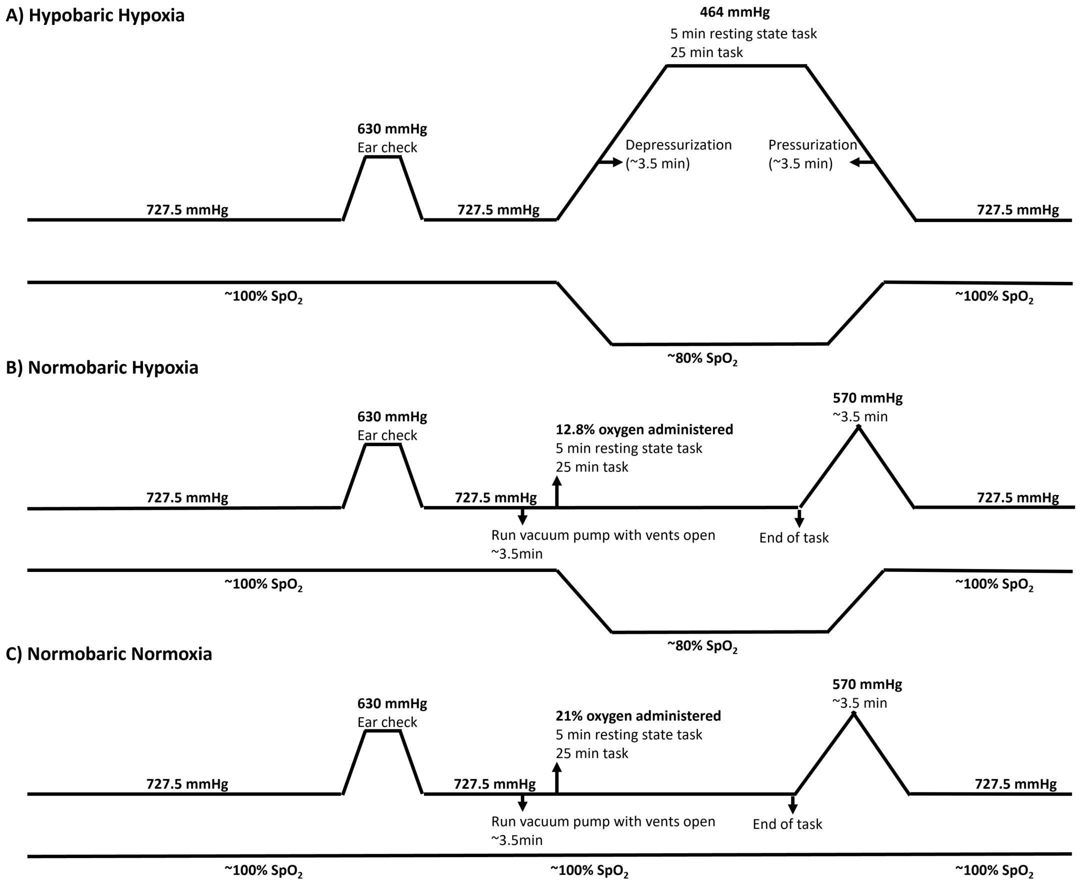

Figure 3. Comparison of the three conditions. A) During the hypobaric hypoxia condition participants first performed a depressurization to 630 mmHg (1554 m) to be certain that they could equalize their ears. Following this we then depressurized the chamber to 464 mmHg (3970 m) and had the participants complete the 5 minutes resting state and 25 minute attention task. Following completion, the chamber was pressurized to 727.5 mmHg (366 m). B) During the normobaric hypoxia condition participants first performed a depressurization to 630 mmHg (1554 m) to be certain that they could equalize their ears. Following this we ran the vacuum pump in the hypobaric chamber for 3.5 minutes with the vents open to simulate the noise of depressurization experienced in the HH conditions. After 3.5 minutes we administer the 12.8% oxygen gas mixture to the participants, and they began

the 5 minutes resting state and 25 minute attention task. Following completion of the task, the hypobaric chamber was depressurized to 570 mmHg (2360 m) and then pressurized to 727.5 mmHg (366 m) in 3.5 min so that the participants felt a pressure change. C) During the normobaric normoxia condition participants first performed a depressurization to 630 mmHg (1554 m) to be certain that they could equalize their ears. Following this ran the vacuum pump in the hypobaric chamber for 3.5 minutes with the vents open to simulate the noise of depressurization experienced in the HH conditions. After 3.5 minutes we administer the 12.8% oxygen gas mixture to the participants, and they began the 5 minutes resting state and 25 minute attention task. Following completion of the task, the hypobaric chamber was depressurized to 570 mmHg (2360 m) and then pressurized to 727.5 mmHg (366 m) in 3.5 minutes so that the participants felt a pressure change.

13 Once you are at the target pressure and ready to begin the experiment, plug in the Portapres, and attach the hose connected to the compressed gas to the end of the modified 2-way non-rebreather valve.

14 Start recording on the EEG data laptop and the physiology recording laptop at the same time (to enable synchronisation of data analyses).

15 When ready, let the participant know that they can begin the experimental recording. In our study the resting state task took five minutes, and the visuospatial attention orienting task took roughly 25 minutes. Although EEG studies with healthy adult participants can sometimes take longer, it is important to balance task time with participant stress, given the lengthy setup and additional stresses incurred by environmental manipulations. If the experimental paradigm allows for breaks, remind the participant to take a couple of seconds to break when the task prompts them to move their feet to lessen the chance of venous pooling in their legs and subsequent potential vasovagal syncope.

16 The inside hypobaric chamber safety attendant will have to watch the SpO_2 and blood pressure monitor to be sure it never drops below the cut-off values. The inside hypobaric chamber safety attendant will also manually adjust the airflow in the back of the hypobaric chamber to match the participants breathing rate, and be certain that the bag never collapses on itself or becomes too full.

17 Once the participant has finished the experiment let the outside hypobaric chamber operator know that you are ready to return to a normal pressure (this will vary depending on hypobaric chamber location). Unplug the blood pressure cuff and remove the mask from the participant and remind them to signal if they have trouble equalizing their ears. The mask is removed so that they can more easily perform a Valsalva maneuver. Offer the participant 100% oxygen for the pressurization if they wish; however, they will have to wear the mask during the entire pressurization. Tell hypobaric chamber operators to begin pressurization. Depending on the condition, this will either be the pressurization to 727.5 mmHg (HH) or the quick depressurization and subsequent pressurization (NH, NN).

18 Once you are finished pressurizing, remove all recording equipment from the participant and help them out of the chamber. Ask the participant which condition they think they performed today and record their answer (as a way to verify the efficacy of the experimental blinding).

19 Repeat the steps for the remaining conditions on separate days.
