# Supplementary material for: A protocol to simultaneously examine cardiorespiratory, cerebrovascular and neurophysiological responses inside a hypobaric chamber
Source: PLoS One. 2024 Oct 24;19(10):e0312622. doi: 10.1371/journal.pone.0312622 (PMC11500867; doi:10.1371/journal.pone.0312622)
Supplement: S2 Text — (DOCX) [file pone.0312622.s002.docx]

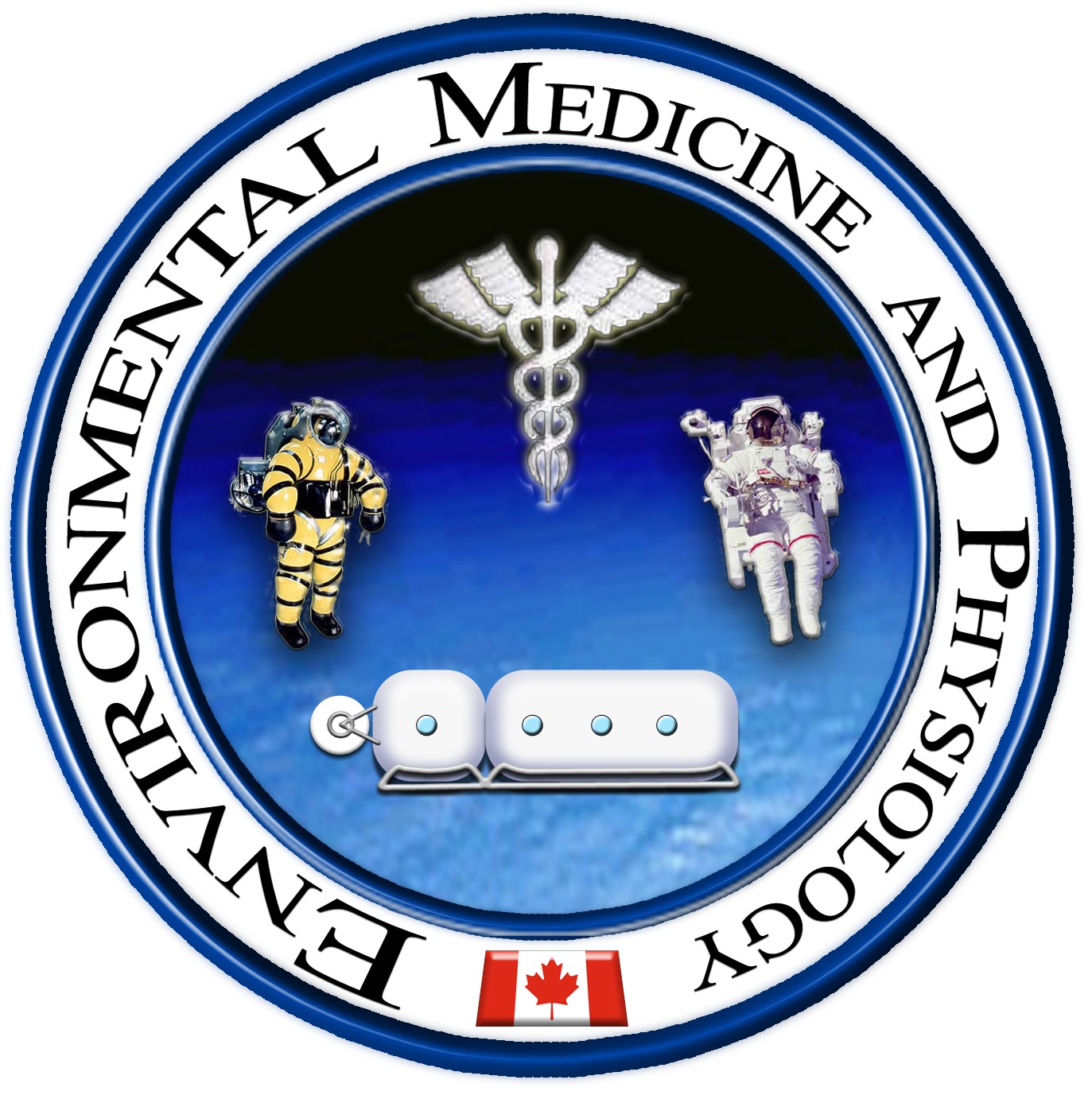

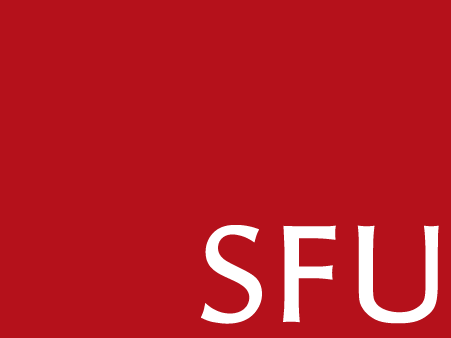


**Medical History Questionnaire**

**To the participant:** The purpose of this medical questionnaire is to find out if the Medical Director should have any concerns he would like to discuss with you prior to you participating in this research study. A positive response to a question does not necessarily disqualify you from participating. A positive response means that there is a pre-existing condition that may affect your safety while participating and should be discussed with the advice of the Medical Director. Please answer each question on your present or past medical history with a YES or NO. If you are not sure please answer YES. If any of these items do not apply to you please answer NO.

Subject number

Age: Sex: Weight Height Have you ever had a collapsed lung?

Do you have a pacemaker? Do you have seizures?

yes no

yes no

yes no

Have you ever had surgery on your eyes or ears? yes no

Have you ever had surgery on your chest? Do you have issues with claustrophobia?

_yes no

yes no

Are you a smoker?

Do you have difficulty clearing your ears? Do you currently have a cold?

Are you or could you be pregnant?

yes no

yes no

yes no

yes no

Do you have a respiratory disease?

yes no

Have you ate/drank in the past couple of hours? yes no

Are you currently taking anti-hypertensive medication

and/or beta blockers? yes no

Do you have anemia? yes no

Do you have a history of fainting? yes no

Do you have a history of not tolerating altitude? yes no

Do you have a history of neurological or yes no

psychiatric illness?

Please list any medical conditions

Please list any prescription medicines you take

Date

Signature

Name
