# Supplementary material for: A protocol to simultaneously examine cardiorespiratory, cerebrovascular and neurophysiological responses inside a hypobaric chamber
Source: PLoS One. 2024 Oct 24;19(10):e0312622. doi: 10.1371/journal.pone.0312622 (PMC11500867; doi:10.1371/journal.pone.0312622)
Supplement: Attachment — Submitted filename: reviewer_comments_plos.docx [file pone.0312622.s003.docx]

Journal requirements:

1. When submitting your revision, we need you to address these additional requirements.

Please ensure that your manuscript meets PLOS ONE's style requirements, including those for file naming. The PLOS ONE style templates can be found at 
<https://journals.plos.org/plosone/s/file?id=wjVg/PLOSOne_formatting_sample_main_body.pdf> and 
<https://journals.plos.org/plosone/s/file?id=ba62/PLOSOne_formatting_sample_title_authors_affiliations.pdf>.

We have made the changes to our headings and author list. We also added supplementary document captions at the end of the manuscript, but before the references.

2. Please provide a complete Data Availability Statement in the submission form, ensuring you include all necessary access information or a reason for why you are unable to make your data freely accessible. If your research concerns only data provided within your submission, please write "All data are in the manuscript and/or supporting information files" as your Data Availability Statement.

We have updated the statement. Due to legal and ethical restriction, data cannot be made publicly available. Data will be made available upon request. Additional published or public analyses would only be permitted with ethics approval for secondary data access. Requests for access to datasets should be made to the corresponding author.

3. We note that Figure(s) 1 and 2 in your submission contain copyrighted images. All PLOS content is published under the Creative Commons Attribution License (CC BY 4.0), which means that the manuscript, images, and Supporting Information files will be freely available online, and any third party is permitted to access, download, copy, distribute, and use these materials in any way, even commercially, with proper attribution. For more information, see our copyright guidelines: <http://journals.plos.org/plosone/s/licenses-and-copyright>.
 
We require you to either (1) present written permission from the copyright holder to publish these figures specifically under the CC BY 4.0 license, or (2) remove the figures from your submission:
 
a. You may seek permission from the original copyright holder of Figure(s) 1 and 2 to publish the content specifically under the CC BY 4.0 license. 
 
We recommend that you contact the original copyright holder with the Content Permission Form (<http://journals.plos.org/plosone/s/file?id=7c09/content-permission-form.pdf>) and the following text:
“I request permission for the open-access journal PLOS ONE to publish XXX under the Creative Commons Attribution License (CCAL) CC BY 4.0 (<http://creativecommons.org/licenses/by/4.0/>). Please be aware that this license allows unrestricted use and distribution, even commercially, by third parties. Please reply and provide explicit written permission to publish XXX under a CC BY license and complete the attached form.”
 
Please upload the completed Content Permission Form or other proof of granted permissions as an ""Other"" file with your submission. 
 
In the figure caption of the copyrighted figure, please include the following text: “Reprinted from [ref] under a CC BY license, with permission from [name of publisher], original copyright [original copyright year].”
 
b. If you are unable to obtain permission from the original copyright holder to publish these figures under the CC BY 4.0 license or if the copyright holder’s requirements are incompatible with the CC BY 4.0 license, please either i) remove the figure or ii) supply a replacement figure that complies with the CC BY 4.0 license. Please check copyright information on all replacement figures and update the figure caption with source information. If applicable, please specify in the figure caption text when a figure is similar but not identical to the original image and is therefore for illustrative purposes only.

We bought a license for BioRender and remade Figure 2 with it.

Figure 1 was commissioned by a co-author (Sherri Ferguson). I have used modified versions of this figure in the past without issue, and this is the first time I am using the original version. I simply stated that it is revised from another paper, which it is.

4. Please include captions for your Supporting Information files at the end of your manuscript, and update any in-text citations to match accordingly. Please see our Supporting Information guidelines for more information: <http://journals.plos.org/plosone/s/supporting-information>.

We have added captions to our supporting information at the end of the manuscript.

[Note: HTML markup is below. Please do not edit.]

Reviewers' comments:

Reviewer's Responses to Questions

**Comments to the Author**


1. Does the manuscript report a protocol which is of utility to the research community and adds value to the published literature?

Reviewer #1: Yes

Reviewer #2: Yes

2. Has the protocol been described in sufficient detail?


To answer this question, please click the link to [protocols.io](http://protocols.io) in the Materials and Methods section of the manuscript (if a link has been provided) or consult the step-by-step protocol in the Supporting Information files.


The step-by-step protocol should contain sufficient detail for another researcher to be able to reproduce all experiments and analyses.

Reviewer #1: Partly

Reviewer #2: Yes

3. Does the protocol describe a validated method?

The manuscript must demonstrate that the protocol achieves its intended purpose: either by containing appropriate validation data, or referencing at least one original research article in which the protocol was used to generate data.

Reviewer #1: Yes

Reviewer #2: No

I did reference two previous research articles where we used the protocol to generate data. When we applied for ethics for those studies we did not state that the data would be available for secondary uses.

4. If the manuscript contains new data, have the authors made this data fully available?

The [PLOS Data policy](http://www.plosone.org/static/policies.action#sharing) requires authors to make all data underlying the findings described in their manuscript fully available without restriction, with rare exception (please refer to the Data Availability Statement in the manuscript PDF file). The data should be provided as part of the manuscript or its supporting information, or deposited to a public repository. For example, in addition to summary statistics, the data points behind means, medians and variance measures should be available. If there are restrictions on publicly sharing data—e.g. participant privacy or use of data from a third party—those must be specified.

Reviewer #1: N/A

Reviewer #2: N/A

**5. Is the article presented in an intelligible fashion and written in standard English?**


PLOS ONE does not copyedit accepted manuscripts, so the language in submitted articles must be clear, correct, and unambiguous. Any typographical or grammatical errors should be corrected at revision, so please highlight any specific errors that need correcting in the box below.

Reviewer #1: Yes

Reviewer #2: Yes

6. Review Comments to the Author

Please use the space provided to explain your answers to the questions above. You may also include additional comments for the author, including concerns about dual publication, research ethics, or publication ethics. (Please upload your review as an attachment if it exceeds 20,000 characters)

Reviewer #1: Method. If a mask leak occurs, the measured end-tidal gases will not be accurate. How is it planned to measure the inspiratory fraction of gases (oxygen, carbon dioxide) in the mask? I mean, in addition to the fact that the internal hypobaric safety assistant will make sure that the reserve bag of respiratory gases never collapses on itself or becomes too full.

Expertise needed to implement protocol “The need for and qualifications of supervising medical personnel depends on the level of the hypoxic stimulus, at a more severe hypoxic dose we recommend having a physician present”. Comment: As this is a research protocol where there may be unexpected events, it is strongly recommended that the physician with expertise in emergency medicine be present at all times.

Thank you for this consideration. We have included this in the revised manuscript


References. 26 out of 44 manuscripts (59%) are older than ten years ago. Where possible, it is recommended that the references be updated.

We have added some updated references.

Reviewer #2: Comments on manuscript “A protocol to simultaneously examine cardiorespiratory, cerebrovascular and neurophysiological responses inside a hypobaric chamber” by Evan A Hutcheon.

The study, authored by Dr Hutcheon, is a proposal for a protocol to investigate neuro-physiological, cerebro-vascular and cardio-respiratory responses in a hypobaric chamber.

Based on their previous experimental experience, the authors describe a setup designed to address several complex physio-pathological questions related to high altitude physiology by integrating data derived from EEG, transcranial Doppler ultrasound, blood pressure, oxygen saturation, end-tidal gases and respiratory rate.


Major comment
The main problem with this study is that it does not produce any real results, although the subject of the study is worthy of attention in view of all the considerations made by the authors. However, real and reproducible data are needed to confirm feasibility and to evaluate physiological responses to hypoxia in different conditions.
I have cited my results from two previous studies where we published with this data.

In addition, the description of the protocol setup has already been published in a dedicated journal, as claimed by the authors.
(<https://protocols.io/view/aprotocol-to-simultaneouslyexamine-cardiorespirac46cyzaw>).

The description I published to protocols.io is a requirement for writing a PloS ONE lab protocol.

Minor comments
What about hypobaric normoxia (e.g. mountaineers breathing oxygen blends at high altitude)?

We did not include this condition as we wanted to explicitly compare normbaric and hypobaric hypoxia, as the literature is sparse as to whether both are equivalent. If they are similar, which has to be validated by more studies, then normobaric hypoxia can be utilized as a surrogate in place of a hypobaric chamber for those studies.

We did include a short section stating that diverse paradigms, such as hypobaric normoxia are possible with our paradigm.

What about simulation of acclimatisation responses? In your protocol, depressurisation is quite fast (a few minutes)?

In theory, acclimatisation protocols are possible; however, these protocols are more complicated and beyond this papers scope. Individuals could stay in the chamber for several days with a commode toilet and food deliveries through the hatch. I have included a short section on how to potentially adapt the paradigm to reflect this.

7. PLOS authors have the option to publish the peer review history of their article ([what does this mean?](https://journals.plos.org/plosone/s/editorial-and-peer-review-process#loc-peer-review-history)). If published, this will include your full peer review and any attached files.


If you choose “no”, your identity will remain anonymous but your review may still be made public.


**Do you want your identity to be public for this peer review?** For information about this choice, including consent withdrawal, please see our [Privacy Policy](https://www.plos.org/privacy-policy).

Reviewer #1: **Yes:** Pasquale Longobardi

Reviewer #2: No
