# Supplementary material for: A protocol to simultaneously examine cardiorespiratory, cerebrovascular and neurophysiological responses inside a hypobaric chamber
Source: PLoS One. 2024 Oct 24;19(10):e0312622. doi: 10.1371/journal.pone.0312622 (PMC11500867; doi:10.1371/journal.pone.0312622)
Supplement: Attachment — Submitted filename: response_to_reviewer.docx [file pone.0312622.s004.docx]

Journal Requirements:

Please review your reference list to ensure that it is complete and correct. If you have cited papers that have been retracted, please include the rationale for doing so in the manuscript text, or remove these references and replace them with relevant current references. Any changes to the reference list should be mentioned in the rebuttal letter that accompanies your revised manuscript. If you need to cite a retracted article, indicate the article’s retracted status in the References list and also include a citation and full reference for the retraction notice.


[Note: HTML markup is below. Please do not edit.]

Reviewers' comments:

Reviewer's Responses to Questions

Comments to the Author


1. Does the manuscript report a protocol which is of utility to the research community and adds value to the published literature?

Reviewer #1: Yes

Reviewer #2: Yes

2. Has the protocol been described in sufficient detail?


To answer this question, please click the link to [protocols.io](http://protocols.io) in the Materials and Methods section of the manuscript (if a link has been provided) or consult the step-by-step protocol in the Supporting Information files.


The step-by-step protocol should contain sufficient detail for another researcher to be able to reproduce all experiments and analyses.

Reviewer #1: Yes

Reviewer #2: Yes

3. Does the protocol describe a validated method?

The manuscript must demonstrate that the protocol achieves its intended purpose: either by containing appropriate validation data, or referencing at least one original research article in which the protocol was used to generate data.

Reviewer #1: Yes

Reviewer #2: Yes

4. If the manuscript contains new data, have the authors made this data fully available?

The [PLOS Data policy](http://c05y1x9s.r.us-east-2.awstrack.me/L0/http:%2F%2Fwww.plosone.org%2Fstatic%2Fpolicies.action%23sharing/1/010f0191e35a47c5-861419a8-001d-472c-9633-522af2413337-000000/f2IHer4Z1tOCs65C8t_CaHGFRsU=176) requires authors to make all data underlying the findings described in their manuscript fully available without restriction, with rare exception (please refer to the Data Availability Statement in the manuscript PDF file). The data should be provided as part of the manuscript or its supporting information, or deposited to a public repository. For example, in addition to summary statistics, the data points behind means, medians and variance measures should be available. If there are restrictions on publicly sharing data—e.g. participant privacy or use of data from a third party—those must be specified.

Reviewer #1: N/A

Reviewer #2: N/A

5. Is the article presented in an intelligible fashion and written in standard English?


PLOS ONE does not copyedit accepted manuscripts, so the language in submitted articles must be clear, correct, and unambiguous. Any typographical or grammatical errors should be corrected at revision, so please highlight any specific errors that need correcting in the box below.

Reviewer #1: Yes

Reviewer #2: Yes

6. Review Comments to the Author

Please use the space provided to explain your answers to the questions above. You may also include additional comments for the author, including concerns about dual publication, research ethics, or publication ethics. (Please upload your review as an attachment if it exceeds 20,000 characters)

Reviewer #1: The manuscript has been diligently revised. A search for similar manuscripts reports for some not cited in your references (see below, ie. Theunissen dated 2022). It remains doubtful that the proposed search protocol is not up to date with the current state of knowledge. I recommend a thorough meta-analysis of the literature.
1) Theunissen S, Balestra C, Bolognési S, Borgers G, Vissenaeken D, Obeid G, Germonpré P, Honoré PM, De Bels D. Effects of Acute Hypobaric Hypoxia Exposure on Cardiovascular Function in Unacclimatized Healthy Subjects: A "Rapid Ascent" Hypobaric Chamber Study. Int J Environ Res Public Health. 2022 Apr 28;19(9):5394. doi: 10.3390/ijerph19095394. PMID: 35564787; PMCID: PMC9102089.
2) Neuhaus C, Hinkelbein J. Cognitive responses to hypobaric hypoxia: implications for aviation training. Psychol Res Behav Manag. 2014 Nov 10;7:297-302. doi: 10.2147/PRBM.S51844. PMID: 25419162; PMCID: PMC4234165

Reviewer #2: The authors of the present paper have responded thoroughly to the reviewers' questions, thereby improving their work.

**Response: Thank you for recommending those two references. They have been added to the manuscript. We have also included the following papers:**

**Rosales AM, Shute RJ, Hailes WS, Collins CW, Ruby BC, Slivka DR. Independent effects of acute normobaric hypoxia and hypobaric hypoxia on human physiology. Sci Rep. 2022 Nov 15;12(1):19570. doi: 10.1038/s41598-022-23698-5. PMID: 36379983; PMCID: PMC9666440.**

**Chroboczek M, Kujach S, Łuszczyk M, Grzywacz T, Soya H, Laskowski R. Acute Normobaric Hypoxia Lowers Executive Functions among Young Men despite Increase of BDNF Concentration. Int J Environ Res Public Health. 2022 Aug 30;19(17):10802. doi: 10.3390/ijerph191710802. PMID: 36078520; PMCID: PMC9518314**

**Hohenauer E, Freitag L, Costello JT, Williams TB, Küng T, Taube W, Herten M, Clijsen R. The effects of normobaric and hypobaric hypoxia on cognitive performance and physiological responses: A crossover study. PLoS One. 2022 Nov 10;17(11):e0277364. doi: 10.1371/journal.pone.0277364. PMID: 36355846; PMCID: PMC9648783.**

**Hohenauer E, Taube W, Freitag L, Clijsen R. Sex differences during a cold-stress test in normobaric and hypobaric hypoxia: A randomized controlled crossover study. Front Physiol. 2022 Sep 23;13:998665. doi: 10.3389/fphys.2022.998665. PMID: 36225301; PMCID: PMC9549379.**

7. PLOS authors have the option to publish the peer review history of their article ([what does this mean?](https://c05y1x9s.r.us-east-2.awstrack.me/L0/https:%2F%2Fjournals.plos.org%2Fplosone%2Fs%2Feditorial-and-peer-review-process%23loc-peer-review-history/1/010f0191e35a47c5-861419a8-001d-472c-9633-522af2413337-000000/nySHJ21w4Q9qVBvJNYatu54XOnQ=176)). If published, this will include your full peer review and any attached files.


If you choose “no”, your identity will remain anonymous but your review may still be made public.


Do you want your identity to be public for this peer review? For information about this choice, including consent withdrawal, please see our [Privacy Policy](https://c05y1x9s.r.us-east-2.awstrack.me/L0/https:%2F%2Fwww.plos.org%2Fprivacy-policy/1/010f0191e35a47c5-861419a8-001d-472c-9633-522af2413337-000000/ZLMrivfDZ0MiYvlYHrq2nn-HVzU=176).

Reviewer #1: Yes: Pasquale Longobardi MD

Reviewer #2: No

[NOTE: If reviewer comments were submitted as an attachment file, they will be attached to this email and accessible via the submission site. Please log into your account, locate the manuscript record, and check for the action link "View Attachments". If this link does not appear, there are no attachment files.]

While revising your submission, please upload your figure files to the Preflight Analysis and Conversion Engine (PACE) digital diagnostic tool, [https://pacev2.apexcovantage.com/](https://c05y1x9s.r.us-east-2.awstrack.me/L0/https:%2F%2Fpacev2.apexcovantage.com%2F/1/010f0191e35a47c5-861419a8-001d-472c-9633-522af2413337-000000/jzmPvfS1czDCvmNYfvm4mbqTO38=176). PACE helps ensure that figures meet PLOS requirements. To use PACE, you must first register as a user. Registration is free. Then, login and navigate to the UPLOAD tab, where you will find detailed instructions on how to use the tool. If you encounter any issues or have any questions when using PACE, please email PLOS at [figures@plos.org](mailto:figures@plos.org). Please note that Supporting Information files do not need this step.
